# Top-Down Influences of Written Text on Perceived Clarity of Degraded Speech

**DOI:** 10.1037/a0033206

**Published:** 2013-06-10

**Authors:** Ediz Sohoglu, Jonathan E. Peelle, Robert P. Carlyon, Matthew H. Davis

**Affiliations:** 1 MRC Cognition and Brain Sciences Unit, Cambridge, UK; 2 MRC Cognition and Brain Sciences Unit and Department of Otolaryngology, Washington University in St. Louis; 3 MRC Cognition and Brain Sciences Unit

**Keywords:** prior knowledge, predictive coding, top-down, vocoded speech, echoic memory

## Abstract

An unresolved question is how the reported clarity of degraded speech is enhanced when listeners have prior knowledge of speech content. One account of this phenomenon proposes top-down modulation of early acoustic processing by higher-level linguistic knowledge. Alternative, strictly bottom-up accounts argue that acoustic information and higher-level knowledge are combined at a late decision stage without modulating early acoustic processing. Here we tested top-down and bottom-up accounts using written text to manipulate listeners’ knowledge of speech content. The effect of written text on the reported clarity of noise-vocoded speech was most pronounced when text was presented before (rather than after) speech (Experiment 1). Fine-grained manipulation of the onset asynchrony between text and speech revealed that this effect declined when text was presented more than 120 ms after speech onset (Experiment 2). Finally, the influence of written text was found to arise from phonological (rather than lexical) correspondence between text and speech (Experiment 3). These results suggest that prior knowledge effects are time-limited by the duration of auditory echoic memory for degraded speech, consistent with top-down modulation of early acoustic processing by linguistic knowledge.

An enduring puzzle is how we understand speech despite sensory information that is often ambiguous or degraded. Whether listening to a speaker with a foreign accent or in a noisy room, we recognize spoken language with accuracy that outperforms existing computer recognition systems. One explanation for this considerable feat is that listeners are highly adept at exploiting prior knowledge of the environment to aid speech perception.

Prior knowledge from a variety of sources facilitates speech perception in everyday listening. Previous studies have shown that lip movements, which typically precede arriving speech signals by ∼150 ms (Chandrasekaran, Trubanova, Stillittano, Caplier, & Ghazanfar, 2009), improve speech intelligibility in noise (Ma, Zhou, Ross, Foxe, & Parra, 2009; Ross, Saint-Amour, Leavitt, Javitt, & Foxe, 2007; Sumby, 1954). Another strong source of prior knowledge is the linguistic context in which an utterance is spoken. Listeners are quicker to identify phonemes located at the ends of words rather than nonwords (Frauenfelder, Segui, & Dijkstra, 1990). For sentences presented in noise, word report is more accurate when the sentences are syntactically and semantically constrained (Boothroyd & Nittrouer, 1988; Kalikow, 1977; Miller & Isard, 1963).

Although the influence of prior knowledge on speech perception is widely acknowledged, there is a longstanding debate about the underlying mechanism. Much of this controversy has centered on one particular effect of prior knowledge: the influence of lexical context on phonological judgments in phonetic categorization and phoneme monitoring tasks (e.g., Frauenfelder et al., 1990; Ganong, 1980; Warren, 1970). One explanation for this phenomenon is that it reflects top-down modulation of phonological processing by higher-level lexical knowledge (McClelland & Elman, 1986; McClelland, Mirman, & Holt, 2006). There are alternative accounts, however, that do not invoke top-down processing. According to these strictly bottom-up accounts, lexical information is combined with phonological information only at a late decision stage where the phonological judgment is formed (Massaro, 1989; Norris, McQueen, & Cutler, 2000).

In the current study, we used a novel experimental paradigm to assess top-down and bottom-up accounts of prior knowledge effects on speech perception. Listeners’ prior knowledge was manipulated by presenting written text before acoustically degraded spoken words. Previous studies have shown that this produces a striking effect on the reported clarity of speech (Sohoglu, Peelle, Carlyon, & Davis, 2012; Wild, Davis, & Johnsrude, 2012; see also Mitterer & McQueen, 2009) that some authors have interpreted as arising from a decision process (Frost, Repp, & Katz, 1988). This is because written text was found to modulate signal detection bias rather than perceptual sensitivity. However, modeling work has shown that signal detection theory cannot distinguish between bottom-up and top-down accounts of speech perception (Norris, 1995; Norris et al., 2000). Hence, an effect of written text on signal detection bias could arise at a late decision stage in a bottom-up fashion or at an early sensory level in a top-down manner. In the current study, we used written text to manipulate both *when* higher-level knowledge becomes available to listeners and the degree of correspondence between prior knowledge and speech input. We will argue that these manipulations more accurately distinguish between top-down and bottom-up accounts.

In the experiments described below, speech was degraded using a noise-vocoding procedure, which removes its temporal and spectral fine structure while preserving low-frequency temporal information (Shannon, Zeng, Kamath, Wygonski, & Ekelid, 1995). Vocoded speech has been a popular stimulus with which to study speech perception because the amount of sensory detail (both spectral and temporal) can be carefully controlled to explore the low-level acoustic factors contributing to speech intelligibility (e.g., Deeks & Carlyon, 2004; Loizou, Dorman, & Tu, 1999; Roberts, Summers, & Bailey, 2011; Rosen, Faulkner, & Wilkinson, 1999; Whitmal, Poissant, Freyman, & Helfer, 2007; Xu, Thompson, & Pfingst, 2005). Furthermore, vocoded speech is widely believed to approximate the information available to deafened individuals who have a cochlear implant (see Shannon et al., 1995). Hence, findings from studies employing vocoded speech not only have implications for understanding the cognitive processes subserving speech perception in normal hearing individuals, but also in the hearing impaired.

When written text is presented before vocoded speech, listeners report that the amount of acoustic degradation is reduced (Wild et al., 2012; see also Goldinger, Kleider, & Shelley, 1999; Jacoby, Allan, Collins, & Larwill, 1988). This suggests that written text modifies listeners’ judgments about the low-level acoustic characteristics of speech. In analogy to the top-down and bottom-up explanations of lexical effects on phonological judgments, there are two mechanisms that could enable prior knowledge from written text to modulate listeners’ judgments about the perceived clarity of vocoded speech. One possibility is that abstract (lexical or phonological) knowledge obtained from text has the effect of modulating early acoustic processing, giving rise to enhanced perceptual clarity (top-down account, see Figure 1a). Alternatively, information from written and spoken sources could be combined at a late decision stage, where the clarity judgment is formed, without modulating early acoustic processing (bottom-up account, see Figure 1b). We now describe the three experiments we have conducted to test these competing accounts of written text effects.[Fig-anchor fig1]

## Experiment 1

Experiment 1 introduces the paradigm that we used to assess the impact of prior knowledge from written text on the perception of vocoded speech. Listeners were presented with vocoded spoken words that varied in the amount of sensory detail, and were asked to rate the perceived clarity of speech. Listeners’ prior knowledge of speech content was manipulated by presenting matching, mismatching, or neutral text before each spoken word. We first characterized the effect of manipulating prior knowledge by asking whether the rated clarity of vocoded speech can be modified not only by the amount of sensory detail conveyed by the vocoder, but also by written text. We assessed both positive and negative effects of prior knowledge on the rated clarity of speech by comparing clarity ratings obtained from matching and mismatching contexts with those from the neutral condition.

One situation that can potentially distinguish between top-down and bottom-up accounts is when knowledge of speech content comes not before but after speech has been heard. This is because there is good evidence that memory for low-level acoustic information (*auditory echoic memory)* has a limited duration. Although estimates of the duration of echoic memory vary depending on the paradigm used (e.g., Crowder & Morton, 1969; Massaro, 1970, 1974; Sams, Hari, Rif & Knuutila, 1993), a consensus has emerged on an early auditory store that preserves unanalyzed acoustic information for around 200–300 ms (see Massaro, 1972; Cowan, 1984; Loveless, Levänen, Jousmäki, Sams, & Hari, 1996).^1^ In contrast, nonsensory information in working memory is widely believed to have a much longer duration (several seconds or longer). It therefore follows that if effects of prior knowledge are attributable to a top-down component that modulates acoustic processing, written text will be less effective in influencing speech perception when presented after the 200−300 ms duration of auditory echoic memory. On the other hand, if effects of prior knowledge arise from a strictly bottom-up mechanism, the influence of written text should be apparent even when presented several seconds after speech. This is because the critical stage of processing in such an account is at the decision stage (where speech information and higher-level knowledge converges). Here, information has been abstracted from the sensory input and hence can be maintained without decay in working memory over several seconds.

To test these two accounts, the critical manipulation in Experiment 1 involved varying the timing of written text and speech so that on some trials text was presented 800 ms before speech onset (the *before* condition) and on other trials text was presented 800 ms after speech onset (the *after* condition). We used monosyllabic spoken words (lasting around 600 ms) in order for acoustic representations of speech to decay by the time of text presentation in the after condition. As described above, this should reduce the influence of text only if attributable to a top-down component.

### Materials and Methods

#### Participants

Nineteen participants were tested after being informed of the study’s procedure, which was approved by the Cambridge Psychology Research Ethics Committee. All were native speakers of English, aged between 18 and 40 years and reported no history of hearing impairment or neurological disease.

#### Stimuli and procedure

A total of 360 monosyllabic words were presented in spoken or written format. The spoken words were 16-bit, 44.1 kHz recordings of a male speaker of southern British English and their duration ranged from 372 to 903 ms (*M* = 600, *SD* = 83).

**Figure 2 fig2:**
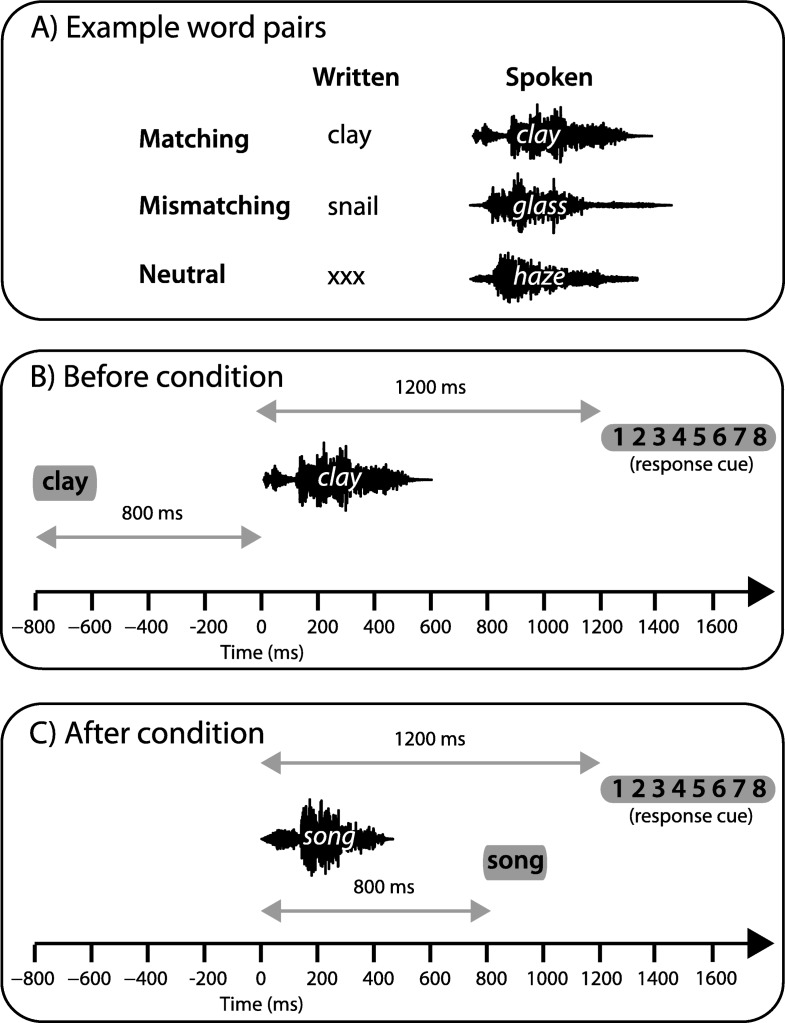
Stimulus characteristics in Experiment 1. A) Example written-spoken word pairs used for matching, mismatching, and neutral conditions. B) Order of events in the before condition. C) Order of events in the after condition.

Written text was presented 800 ms before or after the onset of each spoken word (see Figure 2). Written text contained a word that was the same (matching) or different (mismatching) to the spoken word, or a string of *x* characters (neutral). Written words for the mismatching condition were obtained by permuting the word list for their spoken form. As a result, each written word in the mismatching condition was also presented as a spoken word and vice versa. Mean string length was equated across conditions. Written text was composed of black lowercase characters presented for 200 ms on a gray background.[Fig-anchor fig2]

The amount of sensory detail in speech was varied using a noise-vocoding procedure (Shannon et al., 1995), which superimposes the temporal envelope from separate frequency regions in the speech signal onto corresponding frequency regions of white noise. This allows parametric variation of spectral detail, with increasing numbers of channels associated with increasing perceptual clarity. Vocoding was performed using a custom Matlab (MathWorks Inc.) script, using 1, 2, 4, 8, or 16 spectral channels logarithmically spaced between 70 and 5,000 Hz. Envelope signals in each channel were extracted using half-wave rectification and smoothing with a second-order low-pass filter with a cut-off frequency of 30 Hz. The overall RMS amplitude was adjusted to be the same across all audio files. Pilot data showed that mean word identification accuracy (across participants) for speech with 2, 4 and 8 channels of sensory detail is 3.41% (*SD* = 1.93), 17.05% (*SD* = 1.98) and 68.18% (*SD* = 2.77), respectively. Identification accuracy for 1 channel and 16 channel speech was not tested because it is known from previous studies that for open-set assessment of word recognition, speech with these amounts of sensory detail are entirely unintelligible and perfectly intelligible, respectively (e.g., Obleser, Eisner, & Kotz, 2008; Sheldon, Pichora-Fuller, & Schneider, 2008a).

Manipulations of written text timing (before/after), congruency (matching/mismatching/neutral) and speech sensory detail (1/2/4/8/16 channels) were fully crossed, resulting in a 2 × 3 × 5 factorial design with 12 trials in each condition. Trials were randomly ordered during each of two presentation blocks of 180 trials. The words assigned to each sensory detail and congruency condition were randomized over participants. Given that words were randomly assigned to each participant, we only report the outcome of standard analyses by participants because analyses by items are unnecessary with randomized or counterbalanced designs (Raaijmakers, Schrijnemakers, & Gremmen, 1999).

Stimulus delivery was controlled with E-Prime 2.0 software (Psychology Software Tools, Inc.). Participants were instructed to rate the clarity of each spoken word on a scale from 1 (*Not clear*) to 8 (*Very clear*). To prompt participants to respond, a response cue consisting of a visual display of the rating scale was presented 1,200 ms after the onset of the spoken word (see Figure 2). Participants used a keyboard to record their response and had no time limit to do so. Subsequent trials began 1,000 ms after participants entered their responses. Prior to the experiment, participants completed a practice session of 30 trials containing all conditions but using a different set of words to those used in the main experiment.

### Results

**Figure 3 fig3:**
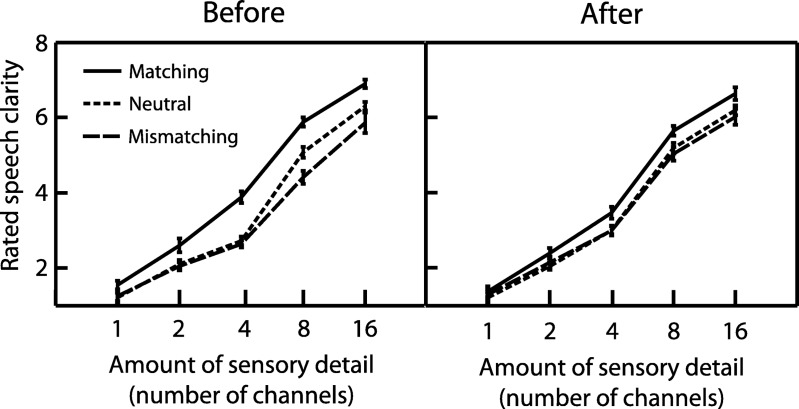
Rated speech clarity in Experiment 1. The provision of increasing sensory detail and prior knowledge from matching text both led to an enhancement in rated speech clarity. The effect of matching text was most pronounced when text appeared before speech onset. Error bars represent SEM across participants corrected for between-participants variability (see Loftus & Masson, 1994).

Ratings of perceived clarity are shown in Figure 3. As expected, a repeated measures ANOVA revealed that increasing sensory detail significantly enhanced clarity ratings (*F*_(4,72)_ = 277, *MS* = 494, η_p_^2^ = .939, *p* < .001). The congruency of written text also enhanced clarity ratings (*F*_(2,36)_ = 43.8, *MS* = 23.2, η_p_^2^ = .709, *p* < .001). Critically, there was a significant interaction between the congruency and timing of written text (*F*_(8,144)_ = 6.78, *MS* = 1.37, η_p_^2^ = .274, *p* < .001), indicating that the effect of written text on clarity ratings was most apparent when written text appeared before speech onset.[Fig-anchor fig3]

**Figure 4 fig4:**
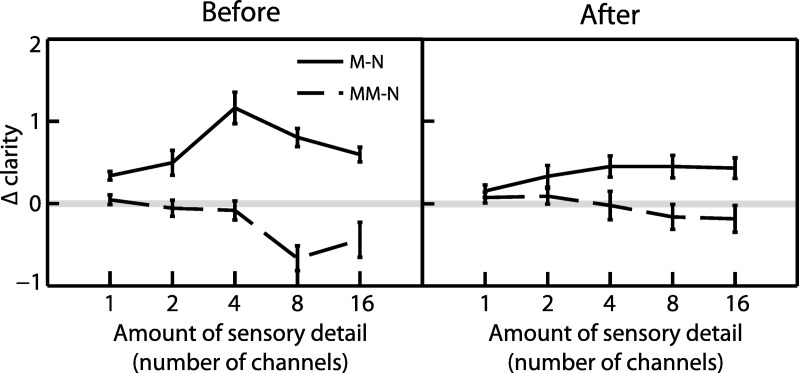
Rated speech clarity in Experiment 1 relative to the neutral condition (Δ clarity). Whereas matching text enhanced speech clarity, mismatching text reduced clarity. Light horizontal gray lines represent no difference in clarity from neutral condition (i.e., zero Δ clarity). Error bars represent SEM across participants corrected for between-participants variability (see Loftus & Masson, 1994). M = Matching; MM = MisMatching; N = Neutral.

To further characterize the influence of written text on clarity ratings, we performed planned contrasts testing for positive effects of matching text and negative effects of mismatching text on clarity relative to neutral text (Δ clarity). As can be seen in Figure 4, matching text significantly enhanced clarity ratings compared with neutral text (*F*_(1,18)_ = 52.9, *MS* = 26.3, η_p_^2^ = .746, *p* < .001). There was also a significant interaction between written text congruency (matching/neutral) and the amount of speech sensory detail (*F*_(4,72)_ = 5.89, *MS* = .859, η_p_^2^ = .246, *p* < .001). We determined the nature of this interaction by conducting a trend analysis on the difference between matching and neutral ratings in the before condition only (i.e., when the effect of written text was most apparent). There was a significant quadratic trend (*F*_(1,18)_ = 14.7, *MS* = 4.18, η_p_^2^ = .450, *p* < .01), indicating that the influence of matching text on clarity ratings was most pronounced for speech with an intermediate amount of sensory detail.[Fig-anchor fig4]

Whereas matching text enhanced clarity ratings, mismatching text significantly reduced clarity ratings relative to neutral text (*F*_(1,18)_ = 7.89, *MS* = 1.76, η_p_^2^ = .305, *p* < .05). This reduction effect was also dependent on the amount of speech sensory detail as there was a significant interaction between written text congruency (mismatching/neutral) and the amount of speech sensory detail (*F*_(4,72)_ = 4.06, *MS* = .843, η_p_^2^ = .184, *p* < .01). As with our previous analysis for matching text, we conducted a trend analysis on the difference between mismatching and neutral ratings in the before condition to examine how the influence of mismatching text varied with sensory detail. In contrast to matching text, there was a significant linear (and not quadratic) trend (*F*_(1,18)_ = 7.90, *MS* = 4.78, η_p_^2^ = .305, *p* < .05), suggesting that the reduction of clarity ratings in response to mismatching text varied in a monotonically increasing manner for speech with a greater amount of sensory detail.

A final analysis examined whether the influence of written text on clarity ratings was apparent for the extreme cases of 1 channel speech (unintelligible without support from written text) and 16 channel speech (highly intelligible). As before, we restricted this analysis to the before condition data. Clarity ratings were significantly greater in the matching relative to mismatching conditions for both 1 channel (one-tailed *t*_(18)_ = 2.82, η^2^ = .306, *p* < .01) and 16 channel speech (one-tailed *t*_(18)_ = 5.74, η^2^ = .647, *p* < .001). This suggests that matching text can enhance ratings of speech clarity over a wide range of conditions (i.e., for unintelligible as well as intelligible speech) even though the extent of this enhancement may differ depending on the amount of sensory detail present (as shown by the congruency by sensory detail interaction). The pattern was different for mismatching text; although there was a significant reduction in clarity ratings in the mismatching relative to neutral conditions for 16 channel speech (one-tailed *t*_(18)_ = −2.04, η^2^ = .188, *p* < .05), there was no significant reduction for 1 channel speech (one-tailed *t*_(18)_ = .938, *p* = .18). This pattern was confirmed by a significant interaction between congruency (mismatching/neutral) and sensory detail (1/16 channels) (*F*_(1,18)_ = 4.59, *MS* = 1.13, η_p_^2^ = .203, *p* < .05). One explanation for the absence of an effect of mismatching text for 1 channel speech is that clarity ratings were at floor for this amount of sensory detail and therefore could not be reduced further by mismatching text.

### Discussion

The results from Experiment 1 demonstrate that prior knowledge of speech content from written text has a measurable effect on the rated clarity of vocoded speech, which replicates previous findings from studies that used a similar paradigm to the one employed here (Goldinger et al., 1999; Jacoby et al., 1988; Sohoglu et al., 2012; Wild et al., 2012).

Our results also suggest that prior knowledge can have both facilitatory and inhibitory effects on speech clarity ratings. Relative to the neutral condition in which prior knowledge of speech content was absent, matching text enhanced clarity ratings, whereas mismatching text reduced ratings. Although the magnitude of these effects varied with the amount of speech sensory detail, a striking finding is that facilitatory effects of written text occurred across the entire range of sensory detail tested (i.e., from entirely unintelligible 1 channel speech to completely intelligible 16 channel speech). This finding is consistent with the study of Frost et al. (1988), who reported that listeners are able to detect correspondence between text and speech even when speech is presented as signal correlated noise (containing only the temporal envelope of speech, i.e., similar to the 1 channel condition here). Such findings indicate that prior knowledge can influence perception with only a minimal amount of sensory information. Nonetheless, for prior knowledge to have adaptive value, its influence must be restricted to auditory signals that contain some speech information to minimize the occurrence of misperceptions. Indeed, Frost et al. also demonstrated that the influence of written text does not extend to white noise that completely lacks speech envelope information.

Finally, the most revealing result for existing accounts of speech perception is our observation that the effects of written text on the rated clarity of vocoded speech were less pronounced when written text was presented 800 ms after speech onset compared to when it was presented 800 ms before speech onset. This finding is readily predicted by a top-down account whereby abstract (lexical or phonological) knowledge obtained from written text modifies lower-level acoustic representations of speech. An important prediction of the model is that for prior knowledge to be effective in modifying speech perception, acoustic representations of speech must persist long enough to permit direct interaction with lexical or phonological representations from written text. Because the majority of the spoken words in Experiment 1 had a duration of ∼600 ms and because previous findings indicate that auditory echoic memory has a limited duration of around 200–300 ms (Cowan, 1984; Loveless et al., 1996; Massaro, 1970, 1974), acoustic representations of speech would have mostly decayed in the condition when text was presented 800 ms after speech onset. As a result, written text would have been less effective in modifying speech clarity. In contrast, it is less obvious how a purely bottom-up account would explain this finding. In such an account, prior knowledge and sensory information are combined at a late decision stage where information has been abstracted from auditory and visual inputs and where information is easily maintained over the period that was required here.

## Experiment 2

In Experiment 2, we sought further evidence that written text influences the rated clarity of vocoded words by modifying auditory echoic traces of speech. As noted above, previous work has estimated the duration of auditory echoic memory to be around 200–300 ms. We therefore manipulated the timing of written text in a finer-grained manner in order to determine whether the duration of auditory echoic memory is precisely reflected in the timecourse of prior knowledge effects. Speech was presented with matching or mismatching text only as this comparison yielded the largest effect of prior knowledge in Experiment 1. The stimulus onset asynchrony (SOA) between written text and speech was varied gradually from −1,600 ms (text before speech onset) to +1,600 ms (text after speech onset) to sample the underlying timecourse of clarity enhancement by prior knowledge.

If the duration of echoic memory is reflected in the timecourse relating clarity enhancement to SOA, two predictions follow. First, in conditions when text is presented before speech onset (negative SOAs), the influence of written text should be maximal and not vary with SOA. This is because in these conditions, abstract lexical or phonological representations from text will be able to modulate acoustic input immediately upon speech arrival and therefore without being constrained by echoic memory decay. Second, in conditions when text is presented after speech onset (positive SOAs), the influence of written text should start to decay only for SOAs longer than 200–300 ms (after echoic memory decay). Note the assumption here is that echoic memory stores acoustic information corresponding to sublexical portions of speech. This is necessarily the case as the 200–300 ms duration of echoic memory is shorter than the typical ∼600 ms duration of monosyllabic words employed in the current study.

### Materials and Method

#### Participants

Fourteen participants were tested after being informed of the study’s procedure, which was approved by the Cambridge Psychology Research Ethics Committee. All were native speakers of English, aged between 18 and 40 years and reported no history of hearing impairment or neurological disease.

#### Stimuli and procedure

The stimuli were similar to those of Experiment 1. A total of 396 monosyllabic words were presented in spoken or written format. The spoken words were 16-bit, 44.1 kHz recordings of the same male speaker for Experiment 1, and their duration ranged from 317 to 902 ms (*M* = 598 ms, *SD* = 81 ms).

Speech was presented with either matching or mismatching text and with 2, 4, or 8 channels of sensory detail. The SOA between speech and written text included the following values (with negative SOAs indicating that text was presented before speech onset): −1,600 ms, −800 ms, −400 ms, −200 ms, −100 ms, 0 ms, +100 ms, +200 ms, +400 ms, +800 ms or +1,600 ms. After a fixed time interval of 2,000 ms relative to the onset of each spoken word, participants were prompted to give their rating of speech clarity. This meant that the period between the onset of written text and presentation of the response cue varied depending on the particular SOA condition. However, in all cases the response cue came after the written text. As in Experiment 1, participants had no time limit with which to give their responses.

Manipulations of congruency (matching/mismatching), speech sensory detail (2/4/8 channels) and SOA were fully crossed, resulting in a 2 × 3 × 11 factorial design with six trials in each condition. Trials were randomly ordered during each of four presentation blocks of 198 trials. For each participant, each of the spoken words appeared twice: once as a matching trial and once as a mismatching trial. The first presentation of each word occurred in the first two blocks of the experiment and the second presentation occurred in the final two blocks. The particular words assigned to each condition were randomized over participants.

Prior to the experiment, participants completed a practice session of 12 trials containing all written text congruency and speech sensory detail conditions presented with SOAs of −800, −100, +100 and +800 ms. For this practice session, a different set of words was used to those in the main experiment. All other details of the stimuli and procedure were the same as in Experiment 1.

### Results

**Figure 5 fig5:**
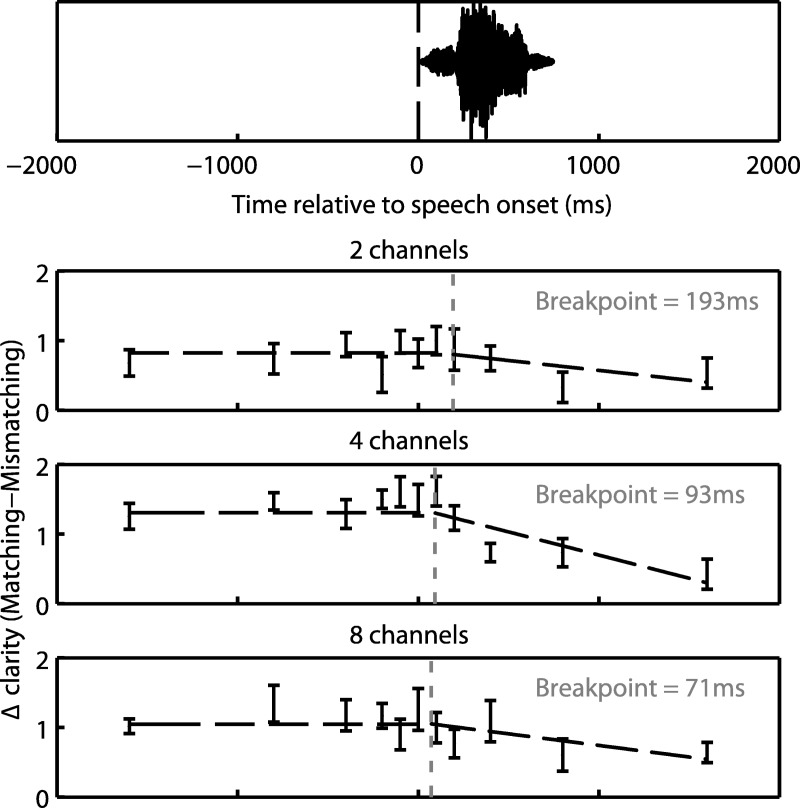
Δ clarity (relative to the mismatching condition) in Experiment 2 as a function of SOA. Each data point represents Δ clarity for the 11 SOA conditions; their *x*-axis locations can be interpreted as the latencies when written text was presented (relative to speech onset, see top panel for an example spoken word). Error bars represent SEM across participants corrected for between-participants variability (see Loftus & Masson, 1994). The black dotted lines represent the breakpoint models given by the mean best-fitting parameters across participants (see main text for details). The vertical gray lines indicate the locations of the mean best-fitting breakpoints.

In order to assess the effect of SOA on ratings of perceived clarity, we conducted a repeated-measures ANOVA on the difference in clarity ratings between speech with matching and mismatching text (Δ clarity), as shown in Figure 5. In addition, we recoded negative and positive SOAs as two separate conditions (before/after) so that factors of written text timing (before/after) and SOA (100/200/400/800/1,600 ms) could be specified. Note that as a result, the 0 ms condition was not included in the ANOVA.[Fig-anchor fig5]

As expected from the findings of Experiment 1, Δ clarity was significantly greater than zero (*F*_(1,13)_ = 22.3, *MS* = 388, η_p_^2^ = .632, *p* < .001) indicating that clarity ratings were enhanced for matching relative to mismatching text. Furthermore, this enhancement was most apparent when text was presented before speech onset (*F*_(1,13)_ = 14.0, *MS* = 8.47, η_p_^2^ = .519, *p* < .01). Critically, there was a significant two-way interaction between written text timing (before/after) and SOA (*F*_(4,52)_ = 3.99, *MS* = 2.23, η_p_^2^ = .235, *p* < .01). Visual inspection of the means suggests that this interaction arose because Δ clarity remained stable with varying SOA when text was presented before speech onset but declined with increasing SOA when text appeared after speech onset. This pattern was confirmed by testing for simple effects of SOA on Δ clarity. These tests revealed no significant effect of SOA on Δ clarity when text was presented before speech onset (*F* < 1). Instead, the effect of SOA was limited to conditions in which text was presented after speech onset (*F*_(4,52)_ = 14.0, *MS* = 3.35, η_p_^2^ = .361, *p* < .001).

We next determined the SOA after which Δ clarity started to decline, which we shall term the *breakpoint*. This problem can be solved by modeling the data in an iterative fashion using a piecewise linear regression procedure (see Hudson, 1966). With this method, the data are modeled as different submodels to reflect a situation in which the relationship between two or more variables (i.e., SOA and Δ clarity) changes at some critical value (i.e., the breakpoint). That critical value is then adjusted until the least-squares error (or other measure of model fit) is minimized. Using this procedure, we fitted two submodels in which Δ clarity was unaffected by SOA before the breakpoint and declined monotonically thereafter, as follows:

′breakpoint<n≤+1600:Δ clarity=mn+c

$$-1600\leq n\leq breakpo\,\mathrm{int}:\Delta \;\text{clarity}={\text{y}}_{\text{b}}$$

where n is the SOA; m and c represent the slope and intercept of a linear-least squares function relating SOA to Δ clarity after the breakpoint; and y_b_ is the value of Δ clarity predicted at/before the breakpoint by this linear least squares function.

We determined the breakpoint that gave the best model fit by systematically varying the breakpoint from 0 to +400 ms and each time computing the root mean square error (RMSE). This was done separately for each sensory detail condition and participant. The model given by the mean best-fitting parameters across participants for each sensory detail condition is shown overlaid onto Figure 5. The mean best-fitting breakpoint across sensory detail conditions and participants was found to be 119 ms. To test whether the breakpoint depended on speech sensory detail, the best-fitting breakpoint for each participant and sensory detail condition was entered into a repeated-measures ANOVA with sensory detail as the within-subjects factor. As the amount of sensory detail increased there was a significant decrease in the breakpoint (*F*_(2,26)_ = 4.38, *MS* = 58809, η_p_^2^ = .252, *p* < .05). A similar analysis was performed for the other parameters of the model: fitted Δ clarity at the breakpoint (quantifying the amount of Δ clarity before it declined) and the best-fitting slope (quantifying the rate of decline). Although the main effect of sensory detail on Δ clarity at the breakpoint was not significant (*F*_(2,26)_ = 1.62, *MS* = .832, η_p_^2^ = .111, *p* = .218), there was a significant quadratic trend (*F*_(1,13)_ = 5.70, *MS* = 11940, η_p_^2^ = .305, *p* < .05) indicating that Δ clarity at the breakpoint was greatest for speech with an intermediate amount (i.e., 4 channels) of sensory detail. Similarly for the best-fitting slope, although the main effect of sensory detail was marginally significant (*F*_(2,26)_ = 2.74, *MS* = 6051, η_p_^2^ = .174, *p* = .083), there was a significant quadratic trend (*F*_(1,13)_ = 5.70, *MS* = 11940, η_p_^2^ = .305, *p* < .05) suggesting that the slope was most negative for speech with an intermediate amount of sensory detail.

A final analysis aimed at determining the impact of speech duration on the enhancement of speech clarity by matching text. A previous study (Hervais-Adelman, Davis, Johnsrude, & Carlyon, 2008) has shown that speech duration is a significant predictor of vocoded speech intelligibility and hence might be expected to influence the relationship between SOA and Δ clarity. Although the spoken words employed in the current experiment were all monosyllabic, they varied in their duration from 317 to 902 ms. This allowed us to compare the bottom quartile of spoken words that had a mean duration of 495 ms (*SD* = 40) to the top quartile that had a mean duration of 701 ms (*SD* = 47). We fitted the same breakpoint model described earlier to the data but no effect of speech duration was found on any of the parameters (Δ clarity at the breakpoint, slope and the breakpoint itself) of the best-fitting model.

### Discussion

In Experiment 2, we confirmed the two predictions of the top-down account. We observed that the influence of written text on speech clarity was maximal and did not vary with SOA when presented before speech onset. It is only when text was presented more than ∼120 ms after speech onset that its influence became progressively less effective. At first glance this might appear to be too early to be explained in terms of a transient auditory echoic memory that lasts ∼200–300 ms. However, this assumes that written text was immediately processed upon presentation, which would not have been the case. Convergent evidence from masked priming (Ferrand & Grainger, 1993; Rastle & Brysbaert, 2006) and neurophysiological (Ashby, Sanders, & Kingston, 2009; Cornelissen et al., 2009; Wheat, Cornelissen, Frost, & Hansen, 2010) studies suggest that we are able to extract phonological information from written text within ∼100 ms of text onset. With a lag of ∼100 ms, the current results suggest that written text was less effective in enhancing speech perception when processed ∼220 ms after speech onset, well within the 200–300 ms range estimated to be the duration of auditory echoic memory. Thus, Experiment 2 provides further evidence consistent with an account in which prior knowledge from written text influences speech perception in a top-down manner by modifying a transient acoustic representation of speech in echoic memory.

The other finding from Experiment 2 is that the SOA at which the influence of written text on rated clarity started to decline (i.e., the breakpoint) decreased for speech that had greater sensory detail. One possible interpretation of this result is that echoic memory is a limited capacity system that decays more rapidly for more complex acoustic information. This interpretation is speculative, as we are not aware of any studies that have systematically explored the relationship between acoustic complexity and the duration of echoic memory. Furthermore, the visual analogue of echoic memory (termed *iconic memory*) is widely believed to be a transient but infinite capacity store (see Loftus, Duncan, & Gehrig, 1992). Hence, if these two forms of sensory memory possess similar characteristics, echoic memory would not be expected to depend on acoustic complexity. An alternative explanation for this finding is the presence of a nonlinear mapping between speech clarity and listeners’ ratings that would have distorted differences in ratings across sensory detail conditions (see Poulton, 1979).

## Experiment 3

In Experiments 1 and 2, we obtained evidence in support of a top-down account of how prior knowledge influences the rated clarity of vocoded speech. According to this account, abstract linguistic information from written text modifies lower-level acoustic processing of speech. In Experiment 3, we asked whether this process is dependent on the lexical or phonological correspondence between text and speech. To address this question, we included an additional congruency condition in which written text partially mismatched with speech by a single phonetic feature in a single segment either at word onset or offset. If effects of prior knowledge depend on perfect lexical correspondence between text and speech, listeners should give this condition the same clarity rating as speech presented with (fully) mismatching text. In contrast, if they depend on phonological correspondence then partial mismatching speech should have an intermediate clarity between matching and mismatching conditions.

In addition to listeners rating the clarity of speech, they were also asked to decide on each trial whether text and speech contained the same word or different words. This second measure enabled us to examine whether the same perceptual effect of written text on clarity ratings could be observed on trials when speech was reported either as matching or mismatching with text.

### Materials and Method

#### Participants

Nineteen participants were tested after being informed of the study’s procedure, which was approved by the Cambridge Psychology Research Ethics Committee. All were native speakers of English, aged between 18 and 40 years and reported no history of hearing impairment or neurological disease.

#### Stimuli and procedure

A total of 576 monosyllabic words were presented in spoken or written format. The spoken words were 16-bit, 44.1 kHz recordings of the same male speaker for Experiments 1 and 2 and their duration ranged from 317 to 903 ms (*M* = 583, *SD* = 77). These items consisted of 144 word pairs selected from the CELEX database (Baayen, Piepenbrock, & Gulikers, 1995) that mismatched either at their onset (e.g., pie/tie) or offset (e.g., lisp/list) segments. Mismatched segments differed in one phonetic dimension only (place of articulation) and included the following pairs: [p] versus [t], [p] versus [k], [t] versus [k], [b] versus [d], [b] versus [g], [d] versus [g], [f] versus [s], [θ] versus [s], and [m] versus [n]. Each word in a pair was randomly assigned to be presented in written or spoken form. Partial mismatching items were obtained directly from these word pairs while matching items were created by randomly selecting one word in a pair and presenting that word in both written and spoken form. Mismatching items were created by randomly shuffling the wordlists for the item pairs.

Matching, partial mismatching or mismatching text was presented 800 ms before the onset of speech. Speech was presented with 2, 4, or 8 channels of sensory detail. Manipulations of congruency (matching/partial mismatching/mismatching) and speech sensory detail (2/4/8 channels) were fully crossed resulting in a 3 × 3 factorial design. We additionally included a factor of item type (onset/offset) to assess the effect of whether partial mismatches occurred at the onset or offset of syllables. Although this comparison was confounded by item differences, any overall difference in speech clarity between onset and offset items should be subtracted out when testing for an interaction between item type and congruency. Furthermore, we would not expect differences between matching and (fully) mismatching trials as a function of onset versus offset. There were 32 trials in each condition that were randomly ordered during each of four presentation blocks of 144 trials. Within each group of onset and offset items, the particular words assigned to each sensory detail and congruency condition were randomized over participants.

Participants were instructed to perform two tasks. As in Experiment 1, participants were prompted 1,200 ms after the onset of each spoken word to rate the clarity of speech on a scale from 1 to 8. Following their response for this clarity rating task, participants were prompted to decide if the spoken word was the same or different to the prior written word. Participants used a keyboard to record their responses for both tasks and had no time limit to do so. Subsequent trials began 1,000 ms after participants entered their responses. Prior to the experiment, participants completed a practice session of 24 trials containing all conditions but using a different set of words to those used in the main experiment. All other details of the stimuli and procedure were the same as in Experiment 1.

### Results

**Figure 6 fig6:**
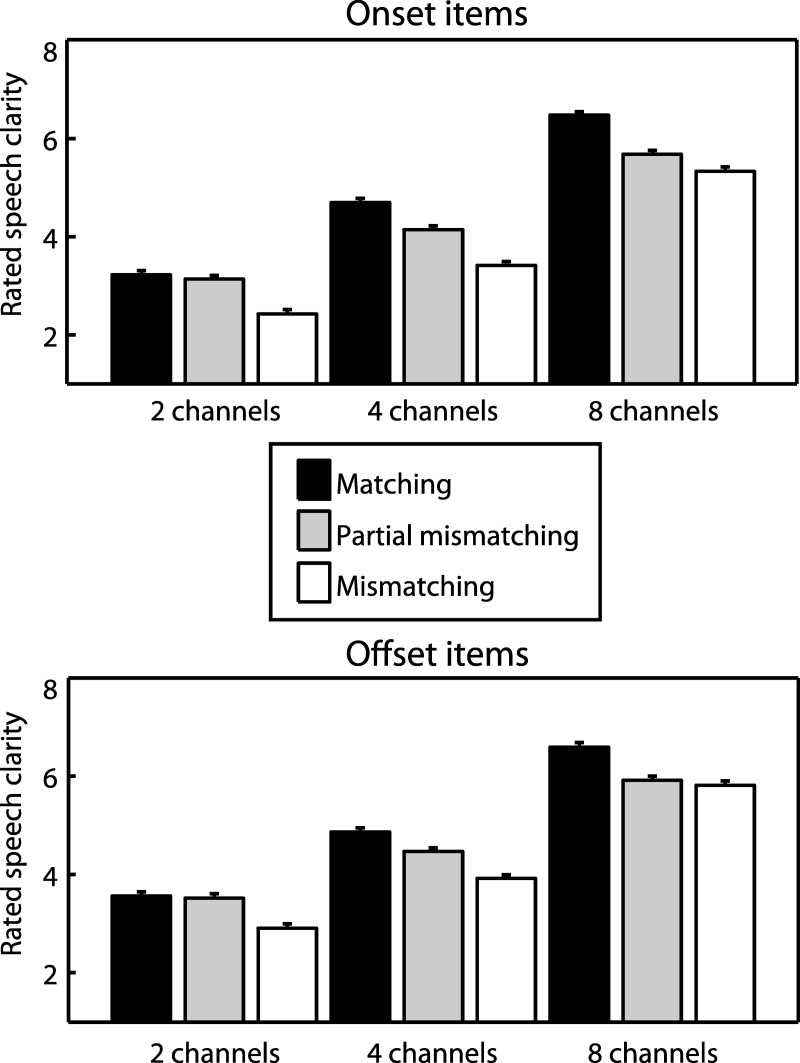
Rated speech clarity in Experiment 3. Speech that was partial mismatching with prior text was rated as being intermediate in clarity between matching and (fully) mismatching speech. Error bars represent SEM across participants corrected for between-participants variability (see Loftus & Masson, 1994).

We first analyzed ratings of perceived clarity in each condition, as shown in Figure 6. We tested whether the partial mismatching condition was rated as being intermediate in clarity between matching and mismatching conditions by conducting two ANOVAs: one that tested for a difference between partial mismatching and matching conditions and one that tested for a difference between partial mismatching and mismatching conditions. For each ANOVA, the factors were congruency, sensory detail (2/4/8 channels) and item type (onset/offset).[Fig-anchor fig6]

Partial mismatching speech was rated as being significantly reduced in clarity relative to the matching condition (*F*_(1,18)_ = 150, *MS* = 10.3, η_p_^2^ = .893, *p* < .001) but significantly greater in clarity relative to the mismatching condition (*F*_(1,18)_ = 45.9, *MS* = 14.6, η_p_^2^ = .718, *p* < .001). Hence, partial mismatching speech was rated as being intermediate in clarity between matching and mismatching conditions. For each comparison, there was a significant interaction between congruency and sensory detail (partial mismatching/matching: *F*_(2,36)_ = 30.8, *MS* = 2.15, η_p_^2^ = .631, *p* < .001; partial mismatching/mismatching: *F*_(2,36)_ = 13.9, *MS* = 1.17, η_p_^2^ = .435, *p* < .001). Visual inspection of the means in Figure 6 suggests that this interaction arose because the intermediate clarity profile of partial mismatching speech became more apparent with an increasing amount of sensory detail. For 2 channel speech that had the least amount of sensory detail, there was no significant difference in clarity ratings between partial mismatching and matching speech (*F*_(1,18)_ = 1.16, *MS* = .088, η_p_^2^ = .061, *p* = .295). This absence of a difference suggests that listeners were misreporting these items as completely matching (an interpretation consistent with our analysis of the same/different task described below). There was, however, a significant difference in clarity ratings between partial mismatching and mismatching conditions for 2 channel speech (*F*_(1,18)_ = 52.4, *MS* = 8.26, η_p_^2^ = .745, *p* < .001), suggesting that listeners were able to detect more extensive mismatches between text and 2 channel speech. All of the above effects were also significant when considering onset and offset items separately.

We next asked whether clarity ratings for the partial mismatching condition depended on whether partial mismatches occurred at the onset or offset of syllables by testing for an interaction between congruency and item type (onset/offset). As mentioned previously, any overall difference in clarity ratings between onset and offset items should cancel out when assessing this interaction. To check that this was the case, we began by including only matching and mismatching conditions in the repeated measures ANOVA. Since these conditions were identical apart from the item lists from which they were drawn, the interaction between congruency and item type should be nonsignificant. However, the interaction was significant (*F*_(1,18)_ = 102, *MS* = 49.4, η_p_^2^ = .850, *p* < .001), thereby preventing us from further assessing the impact of syllable position on perception of partial mismatching speech.^2^ Visual inspection of the means revealed that this interaction arose from a reduction in the difference between matching and mismatching conditions for offset items. As a possible explanation for this unforeseen result, we note that offset items were rated as having significantly greater clarity than onset items (*F*_(1,18)_ = 99.7, *MS* = 9.56, η_p_^2^ = .847, *p* < .001). As previously shown in Experiment 1, the effect of written text on clarity ratings depends on the inherent intelligibility of the speech signal. If this inherent intelligibility changes, whether from increased speech sensory detail, or in the current context, from acoustic or linguistic properties of the items themselves, the effect of written text on clarity ratings will also change. Hence, for previous and subsequent analyses, we do not compare onset and offset conditions directly. To ensure that the remaining (congruency and sensory detail) effects were present for both types of item, we report the results from ANOVAs that average over onset and offset items and also from separate ANOVAs on each item type (unless stated otherwise).

**Figure 7 fig7:**
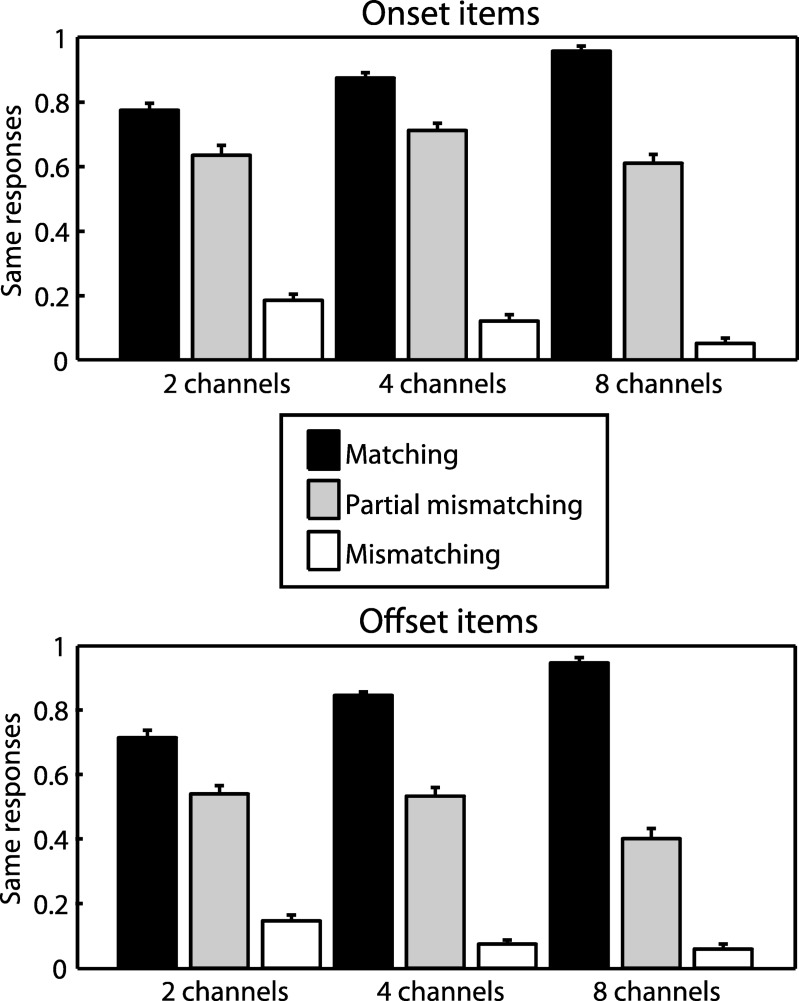
Proportion of responses in which participants in Experiment 3 reported speech and text to contain the same or different words. The proportion of “same” responses to speech that was partial mismatching with prior text was intermediate between matching and (fully) mismatching speech. Error bars represent SEM across participants corrected for between-participants variability (see Loftus & Masson, 1994).

We next analyzed listeners’ responses in the same/different task by computing the proportion of trials in which listeners responded with the “same” judgment and hence reported speech as matching with text. As with our previous ratings analysis, the data were entered into two separate ANOVAs that compared partial mismatching with matching and with mismatching contexts (again including factors of sensory detail and item type). As shown in Figure 7, the proportion of “same” responses made for the partial mismatching condition was significantly lower than the matching condition (*F*_(1,18)_ = 169, *MS* = 4.46, η_p_^2^ = .904, *p* < .001) but significantly higher than the mismatching condition (*F*_(1,18)_ = 330, *MS* = 12.3, η_p_^2^ = .948, *p* < .001). This pattern parallels the clarity profile obtained earlier and indicates that the proportion of “same” responses closely followed the amount of phonological correspondence between text and speech. There was also a significant interaction between congruency and sensory detail for each comparison (partial mismatching vs. matching: *F*_(2,36)_ = 63.7, *MS* = .425, η_p_^2^ = .780, *p* < .001; partial mismatching vs. mismatching: *F*_(2,36)_ = 7.97, *MS* = .054, η_p_^2^ = .307, *p* < .001), indicating that listeners’ accuracy in detecting the correspondence between text and speech improved with increasing amount of sensory detail (i.e., they made more “same” responses in the matching condition and fewer “same” responses in the partial mismatching and mismatching conditions). For 2 channel speech that had the least amount of sensory detail, the proportion of “same” responses for the partial mismatching condition (averaged over onset and offset items) was significantly above the value of 0.5 that would be expected by chance in the absence of a response bias (two-tailed *t*_(18)_ = 2.23, η^2^ = .216, *p* < .05). The proportion of “same” responses was also significantly above chance for 4 channel speech (two-tailed *t*_(18)_ = 3.91, η^2^ = .459, *p* = .001) but not for 8 channel speech (two-tailed *t*_(18)_ = .158, η^2^ = .001, *p* = .876). This suggests that for the 2 channel and 4 channel conditions, not only were listeners unable to detect partial mismatches between text and speech but also were systematically misreporting these items as completely matching. All of the above effects were also significant when considering onset and offset items separately.[Fig-anchor fig7]

Our final analysis established whether the intermediate clarity profile of partial mismatching speech was apparent on the basis of single trials conditioned according to whether listeners reported speech and text to contain the same or different words. This would rule out the possibility that the intermediate clarity profile emerged as a result of averaging across trials: listeners could have reported partial mismatching speech as completely matching (and therefore very clear) on some trials and completely mismatching (and therefore very unclear) on other trials. Hence, for the following analysis, listeners’ judgments of speech clarity were classified according to their responses in the same/different task so that an additional factor of response type (same/different) could be included in repeated measures ANOVA. Only the 4 channel speech conditions were analyzed, as these were the conditions in which the intermediate clarity profile was most apparent and performance in the same/different task was significantly different from chance. Furthermore, the data were averaged over onset and offset conditions to ensure a sufficient number of trials in each condition. Participants who made fewer than three responses in any condition were excluded from this analysis, leaving 16 participants in the resulting dataset. As with our previous analysis, separate ANOVAs were conducted to compare partial mismatching with matching and mismatching conditions.

**Figure 8 fig8:**
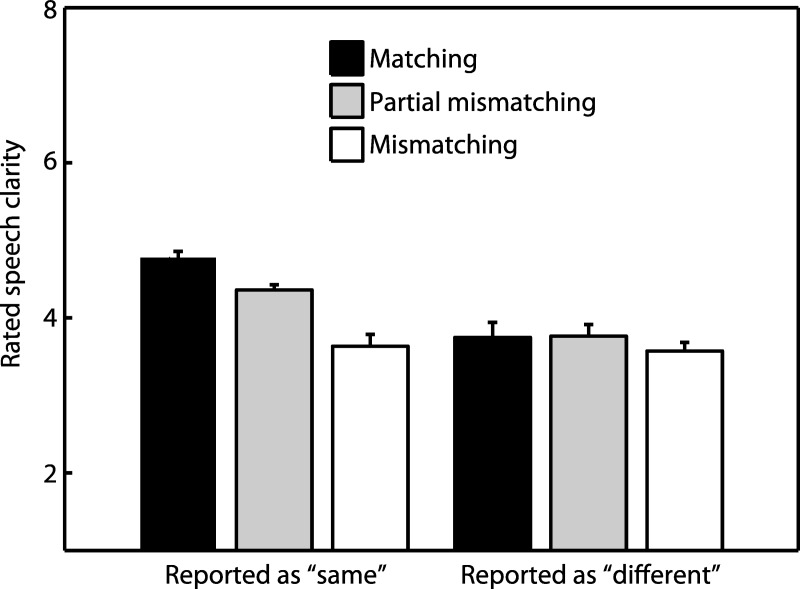
Rated speech clarity in Experiment 3, conditioned on whether participants reported speech and text to contain the same word or different words. The intermediate clarity profile observed previously (i.e., when collapsing across “same” and “different” judgments, as in Figure 6) is also obtained when considering only “same” trials (and hence cannot be attributed to averaging across “same” and “different” trials; see main text for details). Error bars represent SEM across participants corrected for between-participants variability (see Loftus & Masson, 1994).

Figure 8 presents mean clarity ratings for each condition in this new analysis that included the factor of response type (same/different). For trials in which listeners responded with the “same” judgment, the partial mismatching condition differed in clarity from both matching (one-tailed *t*_(15)_ = −8.52, η^2^ = .829, *p* < .001) and mismatching (one-tailed *t*_(15)_ = 4.14, η^2^ = .829, *p* < .001) conditions. Hence, fine-grained differences in listeners’ subjective experience appear to be present even when there are no differences in their final objective report. For “different” trials the pattern changed since although there was a marginally significant difference in clarity ratings between partial mismatching and mismatching conditions (one-tailed *t*_(15)_ = 1.36, η^2^ = .829, *p* = .097), there was no significant difference between partial mismatching and matching conditions (one-tailed *t*_(15)_ = .062, η^2^ = .829, *p* = .476). However, this changed pattern for “different” trials was not supported by a significant interaction between congruency (partial mismatching/matching) and response type (same/different) (*F*_(1,15)_ = 1.91, *MS* = .770, η_p_^2^ = .113, *p* = .187). In summary, this analysis confirms that the intermediate clarity profile observed previously on the basis of clarity ratings alone was also present on trials in which speech was always reported as matching with text and hence cannot be attributed to averaging ratings across trials.[Fig-anchor fig8]

### Discussion

In Experiment 3, we have demonstrated that the magnitude of written text influence on speech clarity varies monotonically with the amount of phonological correspondence between text and speech; the clarity rating obtained for speech presented after partial mismatching text was intermediate between that of matching and mismatching conditions. Thus, effects of written text depend on the phonological (and not lexical) correspondence between text and speech. This phonological interaction between text and speech may arise directly, at a phonological level of processing, or indirectly, between lexical units of representation that share component segments (see Luce & Pisoni, 1998).

The above pattern was also present on trials in which speech was always reported as matching with text and, hence, cannot be an effect attributable to averaging clarity ratings across trials in the matching and mismatching conditions. For that analysis, one might ask why there should have been any differences in clarity given that speech was always reported as matching with text? One possibility is that such differences in subjective experience reflect listeners’ certainty as to the correspondence between text and speech. This result illustrates the utility of using the subjective measure of clarity to probe speech perception as it reveals fine-grained differences in listeners’ subjective experience despite the absence of differences in their final objective report.

Another finding from Experiment 3 that deserves comment is that listeners were systematically responding with the “same” judgment when text partially mismatched with 2 channel and 4 channel speech. This indicates that the level of speech degradation in these conditions was high enough that prior knowledge from written text resulted in listeners misreporting speech as having matched with text. This finding is consistent with a number of previous studies showing that prior knowledge can sometimes have inhibitory effects on perception (Król & El-Deredy, 2011; Samuel, 1981). For example, Samuel (1981) presented listeners with spoken words in which one of the constituent phonemes was replaced by noise; a condition known to result in listeners hearing the missing speech sound (the *phoneme restoration effect*, Warren, 1970). In another condition, the phoneme was intact but had noise added to it. Samuel used signal detection theory to show that listeners’ sensitivity to perceptual differences between these two conditions was worse in lexical contexts that strongly supported the presence of the phoneme (e.g., in words rather than nonwords). Hence, it appears that lexical knowledge in this case was unhelpful to performance on the task as it resulted in both conditions appearing to contain noisy phonemes. Here we have shown that strong but misleading prior knowledge can result in listeners misreporting the correspondence between spoken and written words.

## General Discussion

Prior knowledge is an important source of information that listeners exploit during perception of degraded speech. Across a series of three experiments, we investigated how prior knowledge from written text alters the rated clarity of vocoded spoken words. We now provide further discussion of the results in relation to existing accounts of speech perception and to previous research that has investigated the perception of vocoded speech.

A key finding from the current research is that the effects of prior knowledge were critically dependent on the precise timing of prior knowledge from written text and speech onset. Over the course of Experiments 1 and 2, we showed that written text was progressively less effective in modifying speech clarity when text was presented more than ∼120 ms after speech onset. As discussed previously, this result suggests that integration of prior knowledge and sensory information occurs “online” as listeners hear speech and is well explained by a top-down account (e.g., TRACE; McClelland & Elman, 1986) in which abstract linguistic information from written text is used to modify a transient echoic memory trace of acoustic information from speech that lasts for around 200–300 ms.

In contrast to the top-down account above, a strictly bottom-up mechanism (e.g., Merge; Norris et al., 2000) should not have been time-limited by the duration of echoic memory because the critical computations in such an account occur at a later decision stage of processing where representations have been abstracted from sensory inputs and hence can be maintained in working memory over a period of several seconds without decay. One might argue that our timing manipulation could have affected the decision mechanism in other ways. For instance, listeners could have given less weight to later arriving information, leading to lower clarity ratings when higher-level knowledge from written text was available after speech onset (see Kiani, Hanks & Shadlen, 2008). However, recent evidence suggests that such biases are not reliable across the population when participants are not time-pressured to make their responses (Tsetsos, Gao, McClelland, & Usher, 2012), as was the case in the current study. Furthermore, the top-down account is also consistent with evidence from recent neuroimaging studies employing a similar paradigm to the current study (discussed below). Therefore, the top-down account remains our favored explanation of the present data.

Similar timing effects to those observed here have previously been reported for written word recognition. Rumelhart and McClelland (1982) showed that forced choice accuracy of letter recognition was reduced when the surrounding word context was visible after (rather than before) presentation of the target letter. In the current study we have argued that such timing effects, in conjunction with consideration of the temporal extent of acoustic and higher-level representations of spoken words, successfully distinguish between top-down and bottom-up accounts of speech perception.

The top-down account we have proposed is also consistent with recent findings from studies that have tracked neural activity during perception of vocoded speech with fMRI (Wild et al., 2012) and neurophysiological recordings (EEG/MEG) (Sohoglu et al., 2012). Both studies observed changes in activity in regions of auditory cortex when prior matching text enhanced speech clarity. In the fMRI study by Wild et al., these changes in activity were observed to occur in the most primary region of auditory cortex, suggesting the involvement of a low-level acoustic stage of processing that would be expected to display the characteristics of an echoic memory trace. In addition to activity changes in auditory cortex, these neuroimaging studies also reported changes in prefrontal regions that have been associated with higher-order phonological processing (Booth et al., 2002; Burton, 2001; Hickok & Poeppel, 2007; Price, 2000; Wheat et al., 2010). Furthermore, in the EEG/MEG study of Sohoglu et al., activity changes in these prefrontal regions were observed to occur prior to changes in lower-level auditory regions. This timing profile is uniquely consistent with a top-down mechanism.

Our account of the current data is reminiscent of proposals that perception is initially stimulus driven and involves a bottom-up sweep of information through a processing hierarchy that maintains a form of transient sensory memory for a limited period (Lamme, 2003; Zylberberg, Dehaene, Mindlin, & Sigman, 2009). According to these accounts, a second phase of top-down processing that originates in higher-order stages of the hierarchy subsequently acts to select and maintain a subset of information from this sensory trace for further processing. For stimuli that are degraded and that do not provide immediate access to higher-order representations (such as phonemes or words), this top-down sweep of processing is primarily guided by a second source of information (such as written text). On the basis of the current results, we propose that it is the ensuing recurrent interactions between acoustic and higher-order linguistic representations of speech that underlie the influence of prior knowledge observed here.

### Differing Forms of Top-Down Processing: Interactive or Predictive?

If a top-down mechanism best accounts for the current data, what precise form does this top-down processing take? We have argued elsewhere on the basis of MEG and EEG findings (Sohoglu et al., 2012) that one influential top-down model of speech perception, TRACE, may not in fact implement the type of top-down processing shown during perception of vocoded speech. This is because TRACE is an interactive-activation model with bidirectional excitatory connections between stages of processing that increase activation of model units in those stages. Such organization would lead to equivalent effects on acoustic-phonetic processing in response to increased sensory detail and the provision of higher-level knowledge from matching text. However, we observed opposite effects of these two manipulations on the magnitude of neural responses in auditory cortex (Sohoglu et al., 2012). We suggested that the form of top-down processing that can account for this result is instead implemented by a class of computational model known as *predictive coding* (Arnal & Giraud, 2012; Friston, 2010; Gagnepain, Henson, & Davis, 2012; Rao & Ballard, 1999). This account employs a form of Bayesian hierarchical inference in which the role of top-down information is to predict the activity at lower levels in the hierarchy. During perception, these top-down predictions are adjusted so that they come to match (as closely as possible) the lower-level activity they seek to predict, thereby minimizing prediction error. Accordingly, listening conditions in which top-down predictions explain a larger portion of sensory activity (such as when speech follows matching text) should result in less error and a reduction in activity, as was observed in auditory cortex. In contrast, when the amount of sensory detail is increased in the absence of any prediction for that sensory information, neural responses should increase, which is again what we observed in auditory cortex. Thus, one possibility is that the provision of lexical or phonological knowledge enables more accurate prediction of lower-level acoustic-phonetic representations of speech. Regardless of the precise nature of the underlying top-down process (i.e., interactive or predictive), what is clear is that a top-down mechanism of some kind appears to best explain the available evidence to date.

### Relationship to Previous Research Investigating Perception of Vocoded Speech

The majority of research that has investigated the perception of vocoded speech has focused on the acoustic factors affecting its intelligibility (e.g., Deeks & Carlyon, 2004; Loizou et al., 1999; Roberts et al., 2011; Rosen et al., 1999; Whitmal et al., 2007; Xu et al., 2005). Relatively few studies have explored the role of higher-level cognitive factors, which has been the approach taken here (e.g., Davis, Johnsrude, Hervais-Adelman, Taylor, & McGettigan, 2005; Stacey & Summerfield, 2007; Sheldon et al., 2008b; Loebach, Pisoni, & Svirsky, 2010). Of these latter studies, particular attention has been paid to how comprehension (e.g., as assessed by word report accuracy) improves over the course of exposure. This improvement in speech comprehension is a form of perceptual learning and contrasts with the more immediate aspects of perception studied here. Perceptual learning is likely to be critically important for postlingually deafened cochlear implant users who have to adapt to the novel sounds delivered by their implant after the device is first switched on or whenever adjusted to test new processing strategies (Moore & Shannon, 2009).

Of particular relevance to the current study is the demonstration that providing listeners with knowledge of speech content enhances the rate of learning of vocoded speech (Davis et al., 2005). Other characteristics of learning are shared with the phenomenon studied here. The provision of higher-level knowledge has maximal effect on the rate of learning when provided before (rather than after) speech presentation (Hervais-Adelman et al., 2008). Furthermore, the effect of learning is to alter representations of speech that have not been completely abstracted from the acoustic input (e.g., acoustic-phonetic or allophonic representations) (Dahan & Mead, 2010; Hervais-Adelman, Davis, Johnsrude, Taylor, & Carlyon, 2011). These findings suggests that the top-down effects of prior knowledge on immediate perception observed here may also contribute to longer-term perceptual learning (for a similar proposal in the context of lexically guided perception of ambiguous phonemes, see Mirman, McClelland, & Holt, 2006). Future investigations are needed to confirm this hypothesis and rule out the possibility that effects of prior knowledge on perception and learning occur via separate mechanisms, as has also been proposed (Norris, McQueen, & Cutler, 2003).

## Conclusion

In the three experiments reported here, we have demonstrated that prior knowledge from written text has a powerful influence on the perception of vocoded speech that is comparable to the effect of changing the physical characteristics of the speech signal. Although written text need not precisely match speech for this influence to occur, it must be presented no later than ∼120 ms after speech onset for maximum effect. These findings suggest that the effects of prior knowledge investigated here arise from top-down modulation of auditory processing for degraded speech. They further suggest a critical role for the timing of top-down and sensory inputs that imposes limits on the conditions that allow transient acoustic information to be successfully modulated by higher-level knowledge.

## Figures and Tables

**Figure 1 fig1:**
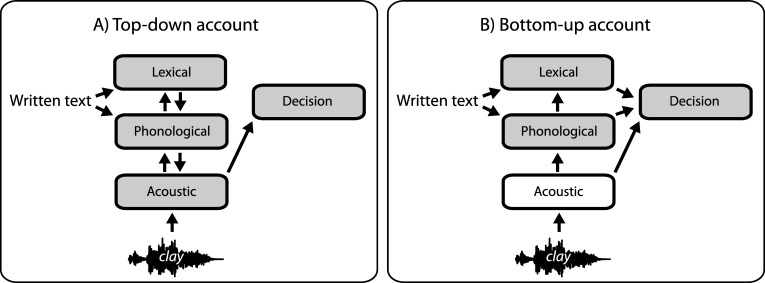
Two competing accounts of how prior lexical or phonological knowledge from written text influences decisions about perceived (acoustic) clarity of vocoded speech. Gray colored boxes indicate the representations that are that potentially modified by written text in each account. A) Top-down account: prior knowledge influences early acoustic processing prior to the decision stage (as in McClelland & Elman, 1986). B) Bottom-up account: prior knowledge and acoustic information are combined at a late decision stage without modulating early acoustic processing (as in Norris et al., 2000).

## Footnotes

1 These authors propose an additional, longer form of auditory storage lasting several seconds. However, the evidence to date suggests that this longer form of auditory storage contains more abstract representations of sound features and sequences and is better described as a form of working (rather than sensory) memory (see Massaro, 1972; Cowan, 1984).

2 We also conducted a linear-mixed effects analysis (Baayen, Davidson, & Bates, 2008) to determine whether this interaction between congruency (matching/mismatching) and item type (onset/offset) would also be significant when taking into account variability in clarity responses attributable to items. Participants and items were entered as random effects, and congruency, sensory detail, and item type (and their interactions) as fixed effects. The interaction was also found to be significant with this analysis suggesting it was general to the population of items tested.

## References

[c1] Arnal L. H., & Giraud A.-L. (2012). Cortical oscillations and sensory predictions. Trends in Cognitive Sciences, 16, 390–398 doi:10.1016/j.tics.2012.05.003 22682813

[c2] Ashby J., Sanders L. D., & Kingston J. (2009). Skilled readers begin processing sub-phonemic features by 80 ms during visual word recognition: Evidence from ERPs. Biological Psychology, 80, 84–94 doi:10.1016/j.biopsycho.2008.03.009 18456383 PMC2677072

[c3] Baayen R. H., Davidson D. J., & Bates D. M. (2008). Mixed-effects modeling with crossed random effects for subjects and items. Journal of Memory and Language, 59, 390–412 doi:10.1016/j.jml.2007.12.005

[c4] Baayen R. H., Piepenbrock R., & Gulikers L. (1995). The CELEX lexical database (Release 2) [CD-ROM]. Philadelphia, PA: Linguistic Data Consortium, University of Pennsylvania, Retrieved from http://www.citeulike.org/user/kids_vr/article/3774173

[c5] Booth J. R., Burman D. D., Meyer J. R., Gitelman D. R., Parrish T. B., & Mesulam M. M. (2002). Functional anatomy of intra- and cross-modal lexical tasks. NeuroImage, 16, 7–22 doi:10.1006/nimg.2002.1081 11969313

[c6] Boothroyd A., & Nittrouer S. (1988). Mathematical treatment of context effects in phoneme and word recognition. Journal of the Acoustical Society of America, 84, 101–114 doi:10.1121/1.396976 3411038

[c7] Burton M. (2001). The role of inferior frontal cortex in phonological processing. Cognitive Science, 25, 695–709 doi:10.1207/s15516709cog2505_4

[c8] Chandrasekaran C., Trubanova A., Stillittano S., Caplier A., & Ghazanfar A. (2009). The natural statistics of audiovisual speech. PLoS Computational Biology, 5(7), e1000436 doi:10.1371/journal.pcbi.1000436 19609344 PMC2700967

[c9] Cornelissen P. L., Kringelbach M. L., Ellis A. W., Whitney C., Holliday I. E., & Hansen P. C. (2009). Activation of the left inferior frontal gyrus in the first 200 ms of reading: Evidence from magnetoencephalography (MEG). PloS One, 4(4), doi:10.1371/journal.pone.0005359 PMC2671164 19396362

[c10] Cowan N. (1984). On short and long auditory stores. Psychological Bulletin, 96, 341–370 doi:10.1037/0033-2909.96.2.341 6385047

[c11] Crowder R. G., & Morton J. (1969). Precategorical acoustic storage (PAS). Perception & Psychophysics, 5, 365–373 doi:10.3758/BF03210660

[c12] Dahan D., & Mead R. L. (2010). Context-conditioned generalization in adaptation to distorted speech. Journal of Experimental Psychology: Human Perception and Performance, 36, 704–728 doi:10.1037/a0017449 20515199

[c13] Davis M. H., Johnsrude I. S., Hervais-Adelman A., Taylor K., & McGettigan C. (2005). Lexical information drives perceptual learning of distorted speech: Evidence from the comprehension of noise-vocoded sentences. Journal of Experimental Psychology: General, 134, 222–241 doi:10.1037/0096-3445.134.2.222 15869347

[c14] Deeks J. M., & Carlyon R. P. (2004). Simulations of cochlear implant hearing using filtered harmonic complexes: Implications for concurrent sound segregation. Journal of the Acoustical Society of America, 115, 1736–1746 doi:10.1121/1.1675814 15101652

[c15] Ferrand L., & Grainger J. (1993). The time-course of phonological and orthographic code activation in the early phases of visual word recognition. Bulletin of the Psychonomic Society, 31, 119–122

[c16] Frauenfelder U. H., Segui J., & Dijkstra T. (1990). Lexical effects in phonemic processing: Facilitatory or inhibitory. Journal of Experimental Psychology: Human Perception and Performance, 16, 77–91 doi:10.1037/0096-1523.16.1.77 2137525

[c17] Friston K. (2010). The free-energy principle: A unified brain theory? Nature Reviews Neuroscience, 11, 127–138 doi:10.1038/nrn2787 20068583

[c18] Frost R., Repp B. H., & Katz L. (1988). Can speech perception be influenced by simultaneous presentation of print? Journal of Memory and Language, 27, 741–755 doi:10.1016/0749-596X(88)90018-6

[c19] Gagnepain P., Henson R. N., & Davis M. H. (2012). Temporal predictive codes for spoken words in auditory cortex. Current Biology, 22, 615–621 doi:10.1016/j.cub.2012.02.015 22425155 PMC3405519

[c20] Ganong W. F. (1980). Phonetic categorization in auditory word perception. Journal of Experimental Psychology: Human Perception and Performance, 6, 110–125 doi:10.1037/0096-1523.6.1.110 6444985

[c21] Goldinger S. D., Kleider H. M., & Shelley E. (1999). The marriage of perception and memory: Creating two-way illusions with words and voices. Memory & Cognition, 27, 328–338 doi:10.3758/BF03211416 10226442

[c22] Hervais-Adelman A., Davis M. H., Johnsrude I. S., & Carlyon R. P. (2008). Perceptual learning of noise vocoded words: Effects of feedback and lexicality. Journal of Experimental Psychology: Human Perception and Performance, 34, 460–474 doi:10.1037/0096-1523.34.2.460 18377182

[c23] Hervais-Adelman A. G., Davis M. H., Johnsrude I. S., Taylor K. J., & Carlyon R. P. (2011). Generalization of perceptual learning of vocoded speech. Journal of Experimental Psychology: Human Perception and Performance, 37, 283–295 doi:10.1037/a0020772 21077718

[c24] Hickok G., & Poeppel D. (2007). The cortical organization of speech processing. Nature Reviews Neuroscience, 8, 393–402 doi:10.1038/nrn2113 17431404

[c25] Hudson D. (1966). Fitting segmented curves whose join points have to be estimated. Journal of the American Statistical Association, 61, 1097–1129 doi:10.1080/01621459.1966.10482198

[c26] Jacoby L. L., Allan L. G., Collins J. C., & Larwill L. K. (1988). Memory influences subjective experience: Noise judgments. Journal of Experimental Psychology: Learning, Memory, and Cognition, 14, 240–247 doi:10.1037/0278-7393.14.2.240

[c27] Kalikow D. N. (1977). Development of a test of speech intelligibility in noise using sentence materials with controlled word predictability. Journal of the Acoustical Society of America, 6, 1337–1351 doi:10.1121/1.381436 881487

[c28] Kiani R., Hanks T. D., & Shadlen M. N. (2008). Bounded integration in parietal cortex underlies decisions even when viewing duration is dictated by the environment. The Journal of Neuroscience, 28, 3017–3029 doi:10.1523/JNEUROSCI.4761-07.2008 18354005 PMC6670720

[c29] Król M. E., & El-Deredy W. (2011). When believing is seeing: The role of predictions in shaping visual perception. The Quarterly Journal of Experimental Psychology, 64, 1743–1771 doi:10.1080/17470218.2011.559587 21824013

[c30] Lamme V. A. F. (2003). Why visual attention and awareness are different. Trends in Cognitive Sciences, 7, 12–18 doi:10.1016/S1364-6613(02)00013-X 12517353

[c31] Loebach J. L., Pisoni D. B., & Svirsky M. A. (2010). Effects of semantic context and feedback on perceptual learning of speech processed through an acoustic simulation of a cochlear implant. Journal of Experimental Psychology: Human Perception and Performance, 36, 224–234 doi:10.1037/a0017609 20121306 PMC2818425

[c32] Loftus G. R., Duncan J., & Gehrig P. (1992). On the time course of perceptual information that results from a brief visual presentation. Journal of Experimental Psychology: Human Perception and Performance, 18, 530–549 doi:10.1037/0096-1523.18.2.530 1593234

[c33] Loftus G. R., & Masson M. E. J. (1994). Using confidence intervals in within-subject designs. Psychonomic Bulletin & Review, 1, 476–490 doi:10.3758/BF03210951 24203555

[c34] Loizou P. C., Dorman M., & Tu Z. (1999). On the number of channels needed to understand speech. Journal of the Acoustical Society of America, 106, 2097–2103 doi:10.1121/1.427954 10530032

[c80] Loveless N., Levänen S., Jousmäki V., Sams M., & Hari R. (1996). Temporal integration in auditory sensory memory: Neuromagnetic evidence. Electroencephalography and Clinical Neurophysiology/Evoked Potentials Section, 100, 220–228 doi:10.1016/0168-5597(95)00271-5 8681863

[c35] Luce P. A., & Pisoni D. B. (1998). Recognizing spoken words: The neighborhood activation model. Ear & Hearing, 19, 1–36 doi:10.1097/00003446-199802000-00001 9504270 PMC3467695

[c36] Ma W. J., Zhou X., Ross L. A., Foxe J. J., & Parra L. C. (2009). Lip-reading aids word recognition most in moderate noise: A Bayesian explanation using high-dimensional feature space. PloS One, 4(3), doi:10.1371/journal.pone.0004638 PMC2645675 19259259

[c37] Massaro D. W. (1970). Preperceptual auditory images. Journal of Experimental Psychology, 85, 411–417 doi:10.1037/h0029712 5489486

[c38] Massaro D. W. (1972). Preperceptual images, processing time, and perceptual units in auditory perception. Psychological Review, 79, 124–45 doi:10.1037/h0032264 5024158

[c39] Massaro D. W. (1974). Perceptual units in speech recognition. Journal of Experimental Psychology, 102, 199–208 doi:10.1037/h0035854 4811941

[c40] Massaro D. W. (1989). Testing between the TRACE model and the fuzzy logical model of speech perception. Cognitive Psychology, 21, 398–421 doi:10.1016/0010-0285(89)90014-5 2758786

[c41] McClelland J. L., & Elman J. L. (1986). The TRACE model of speech perception. Cognitive Psychology, 18, 1–86 doi:10.1016/0010-0285(86)90015-0 3753912

[c42] McClelland J. L., Mirman D., & Holt L. L. (2006). Are there interactive processes in speech perception? Trends in Cognitive Sciences, 10, 363–369 doi:10.1016/j.tics.2006.06.007 16843037 PMC3523348

[c43] Miller G. A., & Isard S. (1963). Some perceptual consequences of linguistic rules. Journal of Verbal Learning and Verbal Behavior, 2, 217–228 doi:10.1016/S0022-5371(63)80087-0

[c44] Mirman D., McClelland J. L., & Holt L. L. (2006). An interactive Hebbian account of lexically guided tuning of speech perception. Psychonomic Bulletin & Review, 13, 958–965 doi:10.3758/BF03213909 17484419 PMC2291357

[c45] Mitterer H., & McQueen J. M. (2009). Foreign subtitles help but native-language subtitles harm foreign speech perception. PloS One, 4(11), doi:10.1371/journal.pone.0007785 PMC2775720 19918371

[c46] Moore D. R., & Shannon R. V. (2009). Beyond cochlear implants: Awakening the deafened brain. Nature Neuroscience, 12, 686–91 doi:10.1038/nn.2326 19471266

[c47] Norris D. (1995). Signal detection theory and modularity: On being sensitive to the power of bias models of semantic priming. Journal of Experimental Psychology: Human Perception and Performance, 21, 935–939 doi:10.1037/0096-1523.21.4.935

[c48] Norris D., McQueen J. M., & Cutler A. (2000). Merging information in speech recognition: Feedback is never necessary. The Behavioral and Brain Sciences, 23, 299–325; discussion 325–370 doi:10.1017/S0140525X00003241 11301575

[c49] Norris D., McQueen J. M., & Cutler A. (2003). Perceptual learning in speech. Cognitive Psychology, 47, 204–238 doi:10.1016/S0010-0285(03)00006-9 12948518

[c50] Obleser J., Eisner F., & Kotz S. A. (2008). Bilateral speech comprehension reflects differential sensitivity to spectral and temporal features. The Journal of Neuroscience, 28, 8116–8123 doi:10.1523/JNEUROSCI.1290-08.2008 18685036 PMC6670773

[c51] Poulton E. C. (1979). Models for biases in judging sensory magnitude. Psychological Bulletin, 86, 777–803 doi:10.1037/0033-2909.86.4.777 482484

[c52] Price C. J. (2000). The anatomy of language: Contributions from functional neuroimaging. Journal of Anatomy, 197, 335–359 doi:10.1046/j.1469-7580.2000.19730335.x 11117622 PMC1468137

[c53] Raaijmakers J. G. W., Schrijnemakers J. M. C., & Gremmen F. (1999). How to deal with “the language-as-fixed-effect fallacy”: Common misconceptions and alternative solutions. Journal of Memory and Language, 41, 416–426 doi:10.1006/jmla.1999.2650

[c54] Rao R. P., & Ballard D. H. (1999). Predictive coding in the visual cortex: A functional interpretation of some extra-classical receptive-field effects. Nature Neuroscience, 2, 79–87 doi:10.1038/4580 10195184

[c55] Rastle K., & Brysbaert M. (2006). Masked phonological priming effects in English: Are they real? Do they matter? Cognitive Psychology, 53, 97–145 doi:10.1016/j.cogpsych.2006.01.002 16554045

[c56] Roberts B., Summers R. J., & Bailey P. J. (2011). The intelligibility of noise-vocoded speech: Spectral information available from across-channel comparison of amplitude envelopes. Proceedings of the Royal Society B: Biological Sciences, 278, 1595–1600 doi:10.1098/rspb.2010.1554 PMC3081737 21068039

[c57] Rosen S., Faulkner A., & Wilkinson L. (1999). Adaptation by normal listeners to upward spectral shifts of speech: Implications for cochlear implants. Journal of the Acoustical Society of America, 106, 3629–3636 doi:10.1121/1.428215 10615701

[c58] Ross L. A., Saint-Amour D., Leavitt V. M., Javitt D. C., & Foxe J. J. (2007). Do you see what I am saying? Exploring visual enhancement of speech comprehension in noisy environments. Cerebral Cortex, 17, 1147–1153 doi:10.1093/cercor/bhl024 16785256

[c59] Rumelhart D. E., & McClelland J. L. (1982). An interactive activation model of context effects in letter perception: II. The contextual enhancement effect and some tests and extensions of the model. Psychological Review, 89, 60–94 doi:10.1037/0033-295X.89.1.60 7058229

[c60] Sams M., Hari R., Rif J., & Knuutila J. (1993). The human auditory sensory memory trace persists about 10 sec: Neuromagnetic evidence. Journal of Cognitive Neuroscience, 5, 363–370 doi:10.1162/jocn.1993.5.3.363 23972223

[c61] Samuel A. G. (1981). Phonemic restoration: Insights from a new methodology. Journal of Experimental Psychology: General, 110, 474–494 doi:10.1037/0096-3445.110.4.474 6459403

[c62] Shannon R. V., Zeng F-G., Kamath V., Wygonski J., & Ekelid M. (1995). Speech recognition with primarily temporal cues. Science, 270, 303–304 doi:10.1126/science.270.5234.303 7569981

[c63] Sheldon S., Pichora-Fuller M. K., & Schneider B. A. (2008a). Effect of age, presentation method, and learning on identification of noise-vocoded words. Journal of the Acoustical Society of America, 123, 476–488 doi:10.1121/1.2805676 18177175

[c64] Sheldon S., Pichora-Fuller M. K., & Schneider B. A. (2008b). Priming and sentence context support listening to noise-vocoded speech by younger and older adults. Journal of the Acoustical Society of America, 123, 489–499 doi:10.1121/1.2783762 18177176

[c65] Sohoglu E., Peelle J. E., Carlyon R. P., & Davis M. H. (2012). Predictive top-down integration of prior knowledge during speech perception. The Journal of Neuroscience, 32, 8443–8453 doi:10.1523/JNEUROSCI.5069-11.2012 22723684 PMC6620994

[c66] Stacey P. C., & Summerfield A. Q. (2007). Effectiveness of computer-based auditory training in improving the perception of noise-vocoded speech. Journal of the Acoustical Society of America, 121, 2923–2935 doi:10.1121/1.2713668 17550190

[c67] Sumby W. H. (1954). Visual contribution to speech intelligibility in noise. Journal of the Acoustical Society of America, 26, 212–215 doi:10.1121/1.1907309

[c68] Tsetsos K., Gao J., McClelland J. L., & Usher M. (2012). Using time-varying evidence to test models of decision dynamics: Bounded diffusion vs. the leaky competing accumulator model. Frontiers in Neuroscience, 6(79). doi:10.3389/fnins.2012.00079 PMC3372959 22701399

[c69] Warren R. M. (1970). Perceptual restoration of missing speech sounds. Science, 167, 392–393 doi:10.1126/science.167.3917.392 5409744

[c70] Wheat K. L., Cornelissen P. L., Frost S. J., & Hansen P. C. (2010). During visual word recognition, phonology is accessed within 100 ms and may be mediated by a speech production code: Evidence from magnetoencephalography. The Journal of Neuroscience, 30, 5229–5233 doi:10.1523/JNEUROSCI.4448-09.2010 20392945 PMC3419470

[c71] Whitmal N. A., Poissant S. F., Freyman R. L., & Helfer K. S. (2007). Speech intelligibility in cochlear implant simulations: Effects of carrier type, interfering noise, and subject experience. Journal of the Acoustical Society of America, 122, 2376–2388 doi:10.1121/1.2773993 17902872

[c72] Wild C. J., Davis M. H., & Johnsrude I. S. (2012). Human auditory cortex is sensitive to the perceived clarity of speech. NeuroImage, 60, 1490–1502 doi:10.1016/j.neuroimage.2012.01.035 22248574

[c73] Xu L., Thompson C. S., & Pfingst B. E. (2005). Relative contributions of spectral and temporal cues for phoneme recognition. Journal of the Acoustical Society of America, 117, 3255–3267 doi:10.1121/1.1886405 15957791 PMC1414641

[c74] Zylberberg A., Dehaene S., Mindlin G. B., & Sigman M. (2009). Neurophysiological bases of exponential sensory decay and top-down memory retrieval: A model. Frontiers in Computational Neuroscience, 3(4). doi:10.3389/neuro.10.004.2009 PMC2659975 19325713

